# Characterization of Enteric Disease in Children by Use of a Low-Cost Specimen Preservation Method

**DOI:** 10.1128/JCM.01703-21

**Published:** 2021-11-18

**Authors:** Amanda K. Debes, Shaoming Xiao, Jie Liu, Allison Shaffer, Paul Scalzo, Etienne Guenou, Landry Beyala, Goura Andre Pascal, Anthony Njimbia Chebe, Hirma Tchio-Nighie, Nafack Sonkeng Sonia, Malathi Ram, David A. Sack, Jerome Ateudjieu

**Affiliations:** a Department of International Health, Johns Hopkins University Bloomberg School of Public Health, Baltimore, Maryland, USA; b University of Virginia, Charlottesville, Virginia, USA; c Meilleur Accès aux Soins de Santé, Yaoundé, Cameroon; d Department of Public Health, Faculty of Medicine and Pharmaceutical Sciences, University of Dschang, Dschang, Cameroon; e Clinical Research Unit, Division of Health Operations Research, Ministry of Public Health, Yaoundé, Cameroon; University of Iowa College of Medicine

**Keywords:** *Shigella*, ETEC, enterics, diagnostics, pediatrics, enteric pathogens, pediatric infectious disease

## Abstract

Diarrhea is a leading cause of death in children under five. Molecular methods exist for the rapid detection of enteric pathogens; however, the logistical costs of storing stool specimens limit applicability. We sought to demonstrate that dried specimens preserved using filter paper can be used to identify diarrheal diseases causing significant morbidity among children in resource-constrained countries. A substudy was nested into cholera surveillance in Cameroon. Enrollment criteria included enrollment between 1 August 2016 and 1 October 2018, age of <18 years, availability of a stool specimen, and having three or more loose stools within 24 h with the presence of dehydration and/or blood. A total of 7,227 persons were enrolled, of whom 2,746 met enrollment criteria and 337 were included in this analysis using the enteric TaqMan array card. Bacterial pathogens were compared to severity of diarrhea, age, and sex, among other variables. One hundred seven were positive for enterotoxigenic Escherichia coli, of which 40.2% (*n* = 43) had heat-labile enterotoxin (LT) and the heat-stable enterotoxin STh, 19.6% (*n* = 21) had LT and the heat-stable enterotoxin STp, and 49.5% (*n* = 53) had LT only. Major colonization factors (CFs) were present in 43.9% of enterotoxigenic E. coli (ETEC)-positive patients. Ninety-six were positive for *Shigella*, of whom 14 (14.6%) reported dysentery. Model-derived quantitative cutoffs identified 116 (34.4%) with one highly diarrhea-associated pathogen and 16 (4.7%) with two or more. *Shigella* and rotavirus were most strongly associated with diarrhea in children with mixed infections. Dried-filter-paper-preserved specimens eliminate the need for frozen stool specimens and will facilitate enteric surveillance and contribute to the understanding of disease burden, which is needed to guide vaccine development and introduction. This study confirms rotavirus, *Shigella*, and ETEC as major contributors to pediatric diarrheal disease in two regions of Cameroon.

## INTRODUCTION

Worldwide, nearly half a million children under 5 years of age die annually from diarrheal diseases, while millions more suffer from multiple episodes throughout early childhood (1). The morbidity associated with pediatric diarrheal disease has long-term consequences, including malnutrition and associated physical and cognitive developmental delays (2). Malnutrition and the resulting stunting increase the risk for a number of chronic disease states later in life (3). Nearly one-third of pediatric diarrheal deaths are due to enterotoxigenic Escherichia coli (ETEC) and *Shigella* (4). In the reanalysis of the Global Enterics Multicenter Study (GEMS), ETEC and *Shigella* were among the top four pathogens, with *Shigella* having the highest rate of pathogen-attributable disease (5), which caused moderate to severe diarrhea (MSD) among children enrolled in the study (6). The use of the TaqMan array card (TAC) demonstrated that *Shigella* and ETEC were two times greater than reported previously using classical microbiological methods of detection (6, 7).

Key stakeholders often lack the disease burden information they need to guide decisions regarding the prioritization of enteric vaccine development (8–11). Classical methods are often not feasible due to lack of laboratory capacity in the remote areas where vaccine trials need to be conducted. PCR methods such as use of the TAC have been shown to improve detection capabilities, allowing nearly 90% of diarrheal episodes to be attributed to a specific pathogen, compared to approximately 50% attribution using classical methods (5, 12). The TAC also helps resolve case attribution in mixed infections, which are common among children under five in low- and middle-income countries (LMICs). In addition, more stringent quantitative cutoffs can be applied, such as “highly diarrhea associated,” applied in the GEMS studies (5).

Freezer storage is required for whole-stool preservation, which entails significant cost, as it is not commonly available in LMICs. Therefore, we validated a modified version of the filter paper specimen preservation method previously developed for cholera surveillance (13) that allows sustained integrity of the nucleic acid in LMICs.

Diarrhea surveillance was implemented in two regions of Cameroon previously identified as areas where cholera is endemic. The surveillance found high rates of cholera-negative diarrhea among persons presenting for the study. All participants were screened by cholera rapid diagnostic test (RDT). The high rates of cholera-negative diarrhea presented the opportunity to evaluate our specimen preservation method as a tool to improve understanding of pathogen-specific diarrheal disease burden. We nested a pediatric subgroup into ongoing cholera surveillance, hypothesizing that ETEC and *Shigella* cause a large proportion of MSD cases, which may be controlled with vaccines currently being developed. This study validated filter paper for specimen preservation at ambient temperature and demonstrated that filter paper specimens can be utilized for PCR detection of diarrheal pathogens to improve understanding of disease burden in various age groups of children in Cameroon. Additionally, our burden estimates resulting from this analysis were consistent with other large-scale pediatric diarrheal studies based on culture and qPCR analysis of fresh or frozen stool samples.

## MATERIALS AND METHODS

### Study design.

The Far North of Cameroon (FNC) is part of the Lake Chad Basin region of Africa. (14), This rural area is unique in that in spite of its isolation it is a crossroads for communication among the bordering countries and, thus, is a center for commercial activities. Food security, water availability, and access to health care are the poorest in Cameroon, likely further exacerbated by the extreme climate (15). These characteristics make this region vulnerable to diarrheal diseases (14).

In contrast, Douala, in the Littoral region, is Cameroon’s largest city. Douala has significant rainfall and a short dry season. The flat relief of the city, the high population density in the slums, and the limited water drainage systems favor floods during the rainy season, contaminating wells and other drinking water sources. As a result, cholera is endemic in Douala (16).

In 2013, diarrheal surveillance began in these two regions, including routinely collecting specimens from acute watery diarrhea (AWD) patients presenting to selected health facilities. Consenting participants completed a questionnaire, including data on access to water, sanitation, and hygiene (WASH), participant and household characteristics, and clinical information, including the presence of blood in stool. Stool specimens were tested via RDT (Crystal-VC, Arkray, India), and 2 drops (∼50 μl) of stool were spotted and preserved on Whatman 903 Protein Saver cards (Sigma-Aldrich, St. Louis, MO) and stored at room temperature (RT) (13). Inclusion criteria for this substudy included enrollment between 8 August 2016 and 1 October 2018, an age of <18 years, having three or more loose stools within 24 h, and presenting with dehydration (determined based on established WHO criteria) and/or blood and/or mucus in stool.

### Sample size calculation.

We hypothesized that 20% of the diarrhea patients would be infected with *Shigella*, with a precision of 10%; we needed to recruit at least 62 children in each age group (0 to 2 years, 3 to 5 years, and 6 to 17 years).

### Nucleic acid extraction.

DNA was extracted from filter paper specimens using Chelex 100 chelating resin (Bio-Rad, Hercules, CA), and the TAC analysis was performed the same day. For each filter paper specimen, half of the filter paper circle was excised and extracted per previously published methods (17). The bacteriophage MS2 and phocine herpesvirus 1 (PhHV) served as external controls. Acid-washed glass beads (Sigma-Aldrich, St. Louis, MO) were added, and the specimen was homogenized for 2 min (Mini Beadbeater-8; BioSpec Products, Bartlesville, OK), prior to the boiling extraction step.

Additional controls included a template-negative control and an extraction blank. Results from any contaminated targets were excluded from the analysis for that extraction batch. The standard curves for each target were created using synthetic targets, plasmids for DNA targets, and *in vitro* transcripts for RNA targets. The standards were run in triplicate and served as the positive controls. To prepare specimens for the TAC analysis, 80 μl of AgPath-ID one-step RT-PCR reagents (Thermo Fisher Scientific, Waltham, MA) was mixed with 20 μl of nucleic acid extract. After loading sample wells, the TAC was centrifuged twice at 1,200 rpm for 1 min, sealed, and loaded into the QuantStudio 12K Flex system (Applied Biosystems, Foster City, CA). The reaction volume was 1 μl for each well, and the cycling conditions were as follows: 1 cycle at 45°C for 20 min, 1 cycle at 95°C for 10 min, and then 40 cycles of 95°C for 15 s and 60°C for 1 min.

### Analysis of the TAC results.

The results were analyzed using the QuantStudio 12K Flex software. Predefined cycle thresholds were set into the software template for each target. For a sample, the negative results of RNA or DNA targets were excluded from the analysis if the quantification cycle (*C_q_*) for the corresponding extrinsic control, MS2 or PhHV, was ≥35. A target was considered negative if the *C_q_* was ≥35.

### Statistical methods.

To compare the demographic information for the three age strata, Student's *t* test was performed using either the pooled or Satterthwaite method depending on whether the variances were equal. Chi-square test or Fisher’s exact test was performed for categorical variables. The prevalence of pathogen target was calculated based on the number of positive results for the target (*C_q_* < 35). The prevalences of highly diarrhea-associated pathogen targets were calculated based on quantities where the point estimate of the odds ratio exceeded 2 (18). The proportions of highly diarrhea-associated pathogen targets were calculated by the ratio of the two prevalences described above. The prevalence and proportion were calculated for the total study population as well as by age strata. The Wilson confidence interval was used for prevalence or proportion. A negative binomial regression model was used to determine if age, gender, region, dysentery, acute diarrhea, or historical antibiotic use was a risk factor for a high number of pathogens in the stool specimen. We defined dysentery, based on the questionnaire, as the presence of “mucus and blood” or “blood” or “watery and blood.” Acute diarrhea was defined as diarrhea in the previous 24 h. A logistic regression model was used to examine whether the covariates described above plus the number of pathogens in the stool were indicative of having a highly diarrhea-associated pathogen. All statistical analyses were conducted in R version 4.0.3 (R Core Team, 2020).

## RESULTS

### Study population.

From August 2016 to October 2018, 7,227 children and adolescents under 18 years of age were enrolled in the cholera surveillance study; 2,746 participants met the inclusion criteria for the pediatric substudy, including 2,234 participants 0 to 2 years old, 277 participants 3 to 5 years old, and 235 participants 6 to 17 years old. We randomly selected 114 (0 to 2 years), 112 (3 to 5 years), and 112 (6 to 17 years) children from these age groups for the nested subgroup analysis (Table 1). We tested 338 specimens using the TAC (5, 18), of which 337 produced valid results. One specimen (3- to 5-year-old age group) was excluded due to poor sample quality. Of the remaining 337, three samples failed to produce valid results for RNA targets, and their RNA targets were removed from the prevalence calculation and the pathogen count analysis. Three additional samples failed to produce valid results for adenovirus; thus, their adenovirus targets were removed from the prevalence calculation, and they were removed from the pathogen count analysis.

**TABLE 1:** Demographic characteristics

Characteristic	Value for group			
	All ages (*n* = 337)	1–2 yrs (*n* = 114)	3–5 yrs (*n* = 111)	6–17 yrs (*n* = 112)
Demographics [no. (%)]				
Gender				
Female	175 (52)	57 (50)	50 (45)	68 (61)
Region				
Douala	199 (59)	59 (52)	69 (62)	71 (63)
Far North	138 (41)	55 (48)	42 (38)	41 (37)
Dysentery	37 (11)	17 (15)	11 (10)	9 (8)
Acute diarrhea	292 (87)	92 (81)	103 (93)	97 (87)
Reported any antibiotics use	315 (93)	110 (96)	106 (95)	99 (88)
No. of pathogens [mean (SD)]	3.1 (2.0)	3.5 (1.9)	2.8 (1.9)	3 (2.2)
No. (%) of individuals with highly diarrhea-associated pathogen^*a*^	132 (40)	40 (36)	45 (41)	47 (42)

a Six samples failed to produce valid results for part of pathogens and were removed from this counting, leaving 311 samples in total.

A secondary aim of this study was to evaluate and adapt a low-cost filter paper preservation method for use with TAC methodologies. Extraction methods were evaluated using spiked stool specimens prior to processing of the participant specimens to ensure that the filter paper-preserved DNA was extracted sufficiently for processing via the TAC (data not shown).

**FIG 1:**
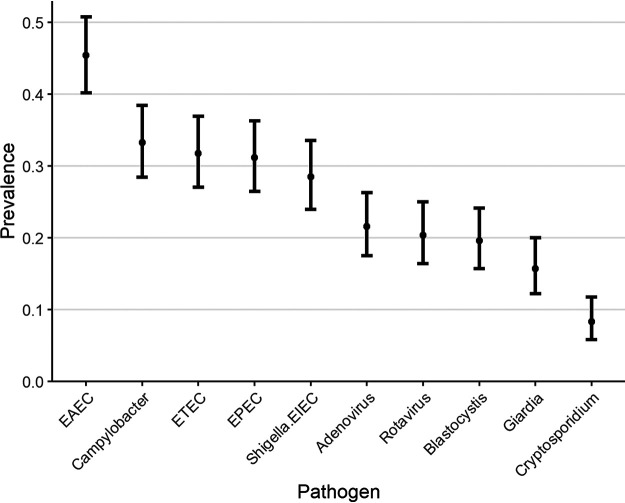
Prevalence of the top 10 pathogens.

Positivity in the qualitative analysis is the presence of any pathogen with a cycle threshold (*C_T_*) of less than 35. We used this threshold to determine which pathogens were present and which were most prevalent among participant stools (Fig. 1). Derived variables are grouped according to defined primer targets (see Table S1 in the supplemental material). Stratifying by age group, enteroaggregative E. coli (EAEC) was most prevalent, with *Shigella* and ETEC being among the five most prevalent pathogens (Table 2).

**TABLE 2:** Prevalence of pathogens (top five) and prevalence of diarrhea-associated pathogens of all samples, stratified by age

Pathogen type and rank	Pathogen (prevalence [95% CI]) in group			
	All ages (*n* = 337)	1–2 yrs (*n* = 114)	3–5 yrs (*n* = 111)	6–17 yrs (*n* = 112)
All pathogens				
1	EAEC (0.45 [0.40–0.51])	EAEC (0.57 [0.49–0.66])	EAEC (0.40 [0.31–0.49])	EAEC (0.39 [0.31–0.49])
2	Campylobacter (0.33 [0.28–0.38])	Campylobacter (0.43 [0.34–0.52])	ETEC (0.32 [0.24–0.41])	Campylobacter (0.33 [0.25–0.42])
3	ETEC (0.32 [0.27–0.37])	EPEC (0.41 [0.33–0.50])	Rotavirus (0.28 [0.21–0.38])	ETEC (0.31 [0.23–0.40])
4	EPEC (0.31 [0.26–0.36])	ETEC (0.32 [0.25–0.42])	*Shigella*/EIEC (0.27 [0.20–0.36])	*Shigella*/EIEC (0.29 [0.22–0.38])
5	*Shigella*/EIEC (0.28 [0.24–0.34])	*Shigella*/EIEC (0.29 [0.21–0.38])	EPEC (0.23 [0.17–0.32])	EPEC (0.28 [0.21–0.38])
Highly diarrhea-associated pathogens				
1	Rotavirus (0.17 [0.13–0.21])	Rotavirus (0.15 [0.09–0.23])	Rotavirus (0.25 [0.18–0.34])	*Shigella*/EIEC (0.23 [0.16–0.32])
2	*Shigella*/EIEC (0.16 [0.12–0.20])	*Shigella*/EIEC (0.13 [0.08–0.21])	*Shigella*/EIEC (0.12 [0.07–0.19])	Rotavirus (0.11 [0.06–0.18])
3	V. cholerae (0.03 [0.02–0.05])	Adenovirus type 40/41 (0.04 [0.01–0.09])	ETEC with STh (0.05 [0.03–0.11])	V. cholerae (0.06 [0.03–0.12])
4	ETEC with STh (0.03 [0.02–0.05])	V. cholerae (0.04 [0.01–0.09])	Salmonella (0.02 [0.00–0.06])	Salmonella (0.03 [0.01–0.08])
5	Adenovirus type 40/41 (0.02 [0.01–0.04])	Salmonella (0.02 [0.00–0.06])	*Cryptosporidium* (0.01 [0.00–0.05])	*Cryptosporidium* (0.03 [0.01–0.08])
6	Salmonella (0.02 [0.01–0.04])	ETEC with STh (0.02 [0.00–0.06])	Adenovirus type 40/41 (0.01 [0.00–0.05])	ETEC with STh (0.02 [0.00–0.06])
7	*Cryptosporidium* (0.01 [0.01–0.03])	*Cryptosporidium* (0.01 [0.01–0.05])	NA^*a*^	Adenovirus type 40/41 (0.02 [0.00–0.06])

a NA, not applicable.

The mean number of pathogens was 3.12 per person, with a range of 0 to 10 pathogens per person (Fig. S2). Ninety-three percent of specimens had detectable pathogens, with nearly 75% having 2 or more pathogens. The negative binomial regression model demonstrated that the expected number of pathogens in the stool for patients meeting the study definition of dysentery was 1.37 times that of those that did not have dysentery (*P* < 0.05). The expected number of pathogens in the stool of those with nonacute diarrhea was 1.38 times that of those with acute diarrhea (*P* < 0.05) (Table S2).

Applying the GEMS quantitative cutoffs for each pathogen, the prevalence of highly diarrhea-associated pathogens did not differ significantly from that of the total population or by age group (Table 3). Forty percent of participants had one or more highly diarrhea-associated pathogen present in their stool. *Shigella* was the most prevalent highly diarrhea-associated pathogen in the older, adolescent age group (6 to 17 years) at 23% and the second most prevalent in the younger two age groups.

**TABLE 3:** Proportion of pathogens that are highly diarrhea associated

Pathogen	No. with pathogen^*a*^		% (95% CI) in group			
	Total	Diarrhea	All ages	1–2 yrs	3–5 yrs	6–17 yrs
Rotavirus	68	56	82 (72–90)	85 (64–95)	87 (71–95)	71 (47–87)
*Shigella*/EIEC	96	54	56 (46–66)	45 (30–62)	43 (27–61)	79 (62–89)
V. cholerae	15	11	73 (48–89)	67 (30–90)	0	88 (53–99)
ETEC with STh	43	10	23 (13–38)	13 (4–38)	50 (25–75)	13 (3–36)
Adenovirus type 40/41	72	7	10 (5–19)	13 (5–30)	5 (8–33)	10 (3–30)
Salmonella	10	7	70 (40–89)	67 (21–98)	50 (15–85)	100 (44–100)
*Cryptosporidium*	28	5	18 (8–36)	7 (1–18)	20 (1–62)	88 (53–99)

a “Total” indicates the total qualitative number of the pathogen present per patient sample analyzed (*C_T_* > 35), and “Diarrhea” is the number of participant samples with pathogens present at highly diarrhea-associated levels.

After adjusting for other covariates, the logistic regression demonstrated that the presence of one or more pathogen in the stool increased the odds of having a highly diarrhea-associated pathogen present by 44% (*P* < 0.05). The odds of having a highly diarrhea-associated pathogen present were higher in males by 63% (*P* < 0.05) and higher for those from Douala, in the Littoral region, by 119% (*P* < 0.05) (Table S3). The overall proportion of pathogens that are highly diarrhea associated demonstrates that of the 96 participants with *Shigella* in their stool, 56% presented with *Shigella* that is highly diarrhea associated (Table 3).

Of the 337 specimens, 96 (28.5%) were positive at any quantity for *Shigella* or enteroinvasive E. coli (EIEC), characterized by the gene *ipaH*. *Shigella*/EIEC was the sixth most common pathogen seen in individuals with dysentery, with 37.8% (14/37) individuals with dysentery testing positive at any quantity. Of participants >5 years of age, 29.5% (*n* = 33) were *Shigella* positive. *Shigella*/EIEC was the most common pathogen associated with dysentery, accounting for 52.9% (9/17) of dysentery cases with an attributable cause and 24.3% (9 of 37) of the total dysentery cases. Genes indicative of Shigella flexneri, including primer targets for any S. flexneri strain, were seen in 59.2% (32 of 54) of all *Shigella*-attributable cases and 46.9% (45 of 96) of all *Shigella* cases in any quantity. Thirty (67%) of these patients were from Douala and 15 (33%) were from the Far North. Shigella sonnei was identified in 20.4% (11 in 54) of all *Shigella*-attributable cases and 12.5% (12/96) of all *Shigella* cases at any quantity; the majority of the patients were from Douala (92%; 11 of 12). Eighty-five percent (82/96) of participants positive for *Shigella* in any quantity did not present with dysentery; further, 83% (45/54) of the attributable cases of *Shigella* infection were nondysenteric.

One hundred seven (32%) were positive in any quantity for ETEC, defined as positive for heat-labile enterotoxin (LT) or the heat-stable enterotoxin STh or STp. Of these, 53 (49.5%) were positive for LT only, 10 (9.3%) were positive for ST only, and 44 (41.1%) were positive for LT and ST. Of those positive for ST, 11 (20.4%) were positive for STp, 33 (61.1%) were positive for STh, and 10 (18.5) were positive for both STp and STh. Of participants >5 years of age, 31.3% (*n* = 35) were ETEC positive. Of these, all experienced watery diarrhea. STh was present in attributable quantities in 10 samples. Neither LT nor STp had quantitative cutoffs available in the literature.

The distribution of ETEC toxin and 6 major colonization factor (CF) types were analyzed. Overall, CFs were present in 43.9% (47 of 107) of ETEC-positive individuals. Coli surface antigen 6 (CS6) was the most common, followed by CFA I, CS2, CS3, and CS1. We detected a CS in 6 patients in whom we did not detect LT or ST (Table S4).

**FIG 2:**
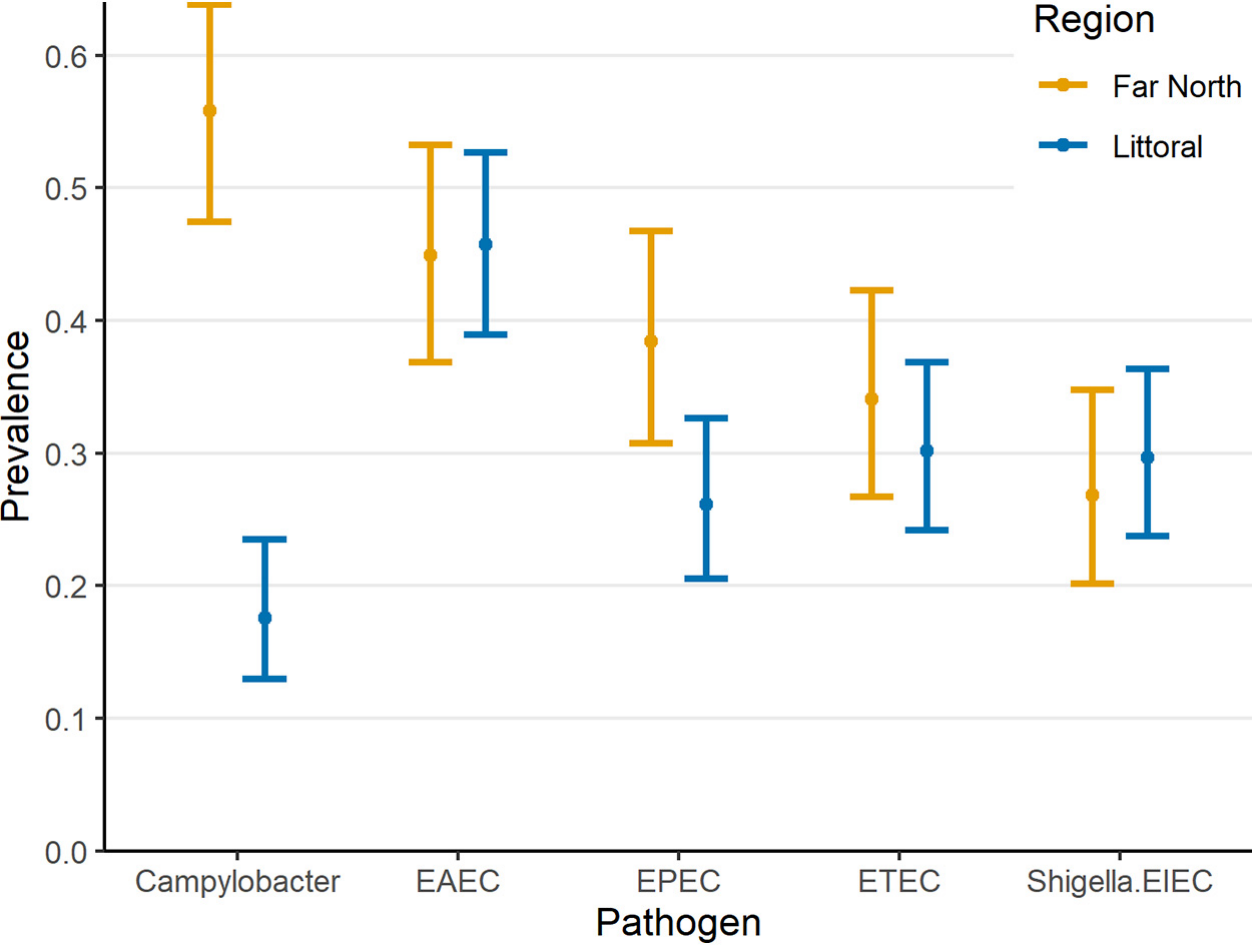
Prevalence of the top 5 pathogens, stratified by region.

Campylobacter was the second most common pathogen detected, present in 33.2% (*n* = 112) of specimens. Sixty-six (58.9%) were positive for Campylobacter jejuni or Campylobacter coli. Despite high presence in the qualitative analysis, Campylobacter did not meet the cutoff to suggest disease attribution. Campylobacter was present in 77 (68.8%) specimens from the Far North and 35 (31.2%) from Douala, a statistically significant difference in prevalence. Figure 2 presents the prevalence of the top 5 pathogens, stratified by region. Seasonality was evaluated but did not demonstrate a significant difference by pathogen or region (data not shown).

**FIG 3:**
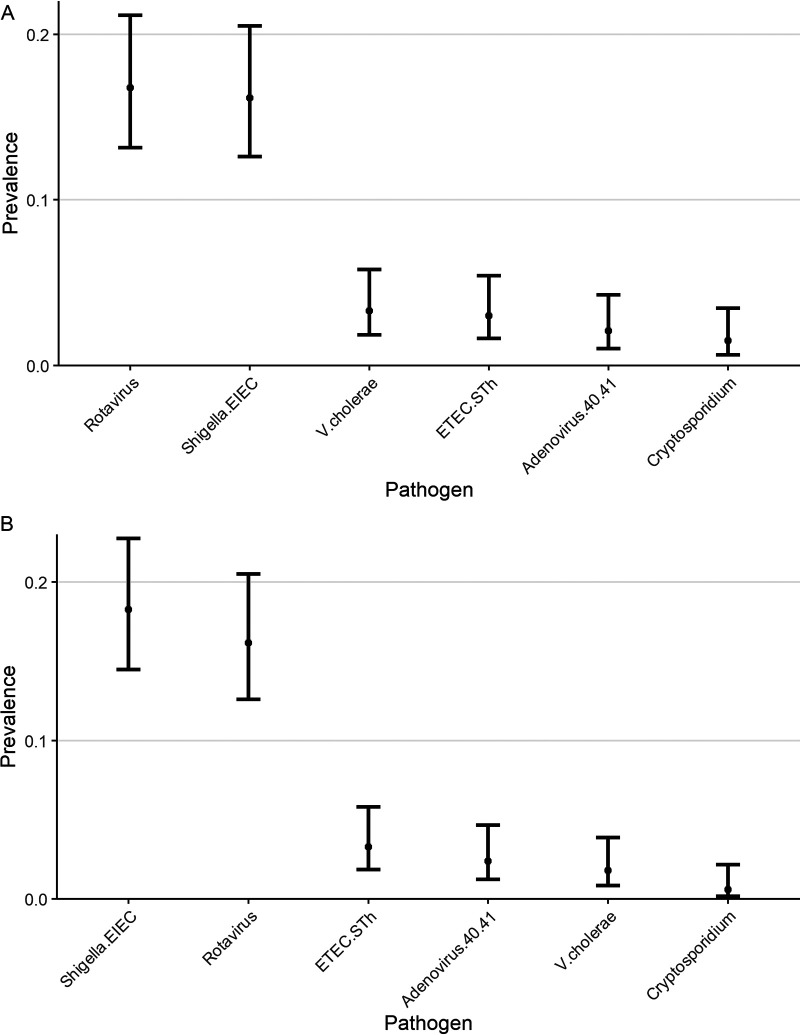
Alignment of our study results (A) in comparison to the prevalence of highly diarrhea-associated pathogens in GEMS and (B) in comparison to the prevalence of highly diarrhea-associated pathogens in the MAL-ED study.

Regardless of which study thresholds were used, comparing GEMS (0 to 5 age strata) and the MAL-ED study (0 to 2 age strata), the results remain similarly aligned (Fig. 3). Per the MAL-ED study, *Shigella* is the most prevalent highly diarrhea-associated pathogen.

## DISCUSSION

This study characterized pediatric diarrheal samples from multiple locations with high rates of diarrhea, where previously there was limited information on the circulating etiologic agents. This study demonstrated that through optimization of nucleic acid extraction from specimens preserved on low-cost filter paper, we can examine stool specimens for a range of enteric and serotype-specific information on several key targets, including enterotoxigenic E. coli (ETEC) and *Shigella*.

We conducted both qualitative and quantitative analyses to assess the primary outcome, confirming our primary hypothesis of high rates of ETEC and *Shigella* in the pediatric population. The top five pathogens demonstrate that *Shigella*, ETEC, and Campylobacter are highly present among the participants. The most common pathogens by age group were similar (Fig. S1 and S2). The regional differences demonstrate the need for further investigation. Importantly, the data demonstrate that the five most prevalent pathogens remain the same regardless of age. Research on the causal pathways contributing to environmental enteropathy, stunting, and other long-term negative health outcomes among infants and young children in LMICs indicates that even asymptomatic colonization with these enteropathogens should be a public health concern, since it may impact growth, cognitive development, and long-term economic productivity (3, 19, 20) Additionally, we found high rates of *Shigella* and ETEC in age groups older than 5 years, confirming the importance of these two pathogens in older children and adolescents (21).

A secondary aim was to use qPCR to determine the quantity of bacterial DNA present and to correlate degrees of clinical illness and case attribution. Since the parent study did not involve the enrollment of controls, we were unable to establish independent cutoffs and applied previously established cutoffs (5). While neither GEMS nor the MAL-ED study had a site in Cameroon, both large studies included sites in sub-Saharan Africa focusing on pediatric populations. We applied the “highly diarrhea-associated quantity” for more stringent cutoffs in our analysis. We were able to identify etiological pathogens for 132 (39.9%) of the samples tested. Further, using the filter paper preservation method, we were successful at this rate in a population where more than 90% had taken antibiotics. Seven of 14 pathogens were attributable quantities; rotavirus was the most commonly present attributable pathogen, followed by *Shigella*/EIEC and ETEC with STh. One hundred sixteen (87.8%) cases had one diarrhea-associated pathogen detected, 15 (11.4%) had two, and 1 (0.8%) had three. Campylobacter and EAEC were two of the most common pathogens found in any quantity, but no cases were attributable to either.

Limitations of our analysis include the lack of controls due to the nested design. Further, we utilized filter paper-preserved specimens which consisted of 50 μl of dried stool specimen. To address these two limitations, we used the GEMS-derived cutoffs, and for simplicity, we applied the more stringent highly diarrhea-associated cutoff values for *C_q_* rather than copy number. Utilizing the GEMS cutoffs to attribute disease with the broad inclusion of bacteria, viruses, and parasites identified several interesting findings for which we hope to build future studies. The filter paper cards were preserved at RT; while we have not previously observed degradation using this method, the TAC used in this analysis looks at both RNA and DNA viruses. Additional research is needed to fully understand the stability of filter paper-preserved specimens stored at various temperatures. Our results do indicate finding similar to those of other studies that used such methods (5, 7, 18, 22).

In conclusion, we were able to utilize specimens preserved on low-cost filter paper to identify diarrheal pathogens prevalent in a pediatric population. We confirmed that ETEC and *Shigella* cause a large proportion of MSD illnesses which may be controlled with vaccines currently being developed. We applied stringent cutoffs to minimize the likelihood of false attribution and improve applicability for vaccine trials. Ongoing trials of current *Shigella* and ETEC vaccines will need validation in additional countries of endemicity (11, 23, 24), and this study suggests that *Shigella* and ETEC are endemic in the pediatric population in Cameroon. The findings from this study support the use of filter paper preservation of stool specimens for PCR confirmation and, therefore, a more accurate understanding of diarrheal disease burden in the pediatric population in Cameroon.
