# Supplementary material for: Characterization of Enteric Disease in Children by Use of a Low-Cost Specimen Preservation Method
Source: J Clin Microbiol. 2021 Nov 18;59(12):e01703-21. doi: 10.1128/JCM.01703-21 (PMC8601215; doi:10.1128/JCM.01703-21)
Supplement: Supplemental file 1 — Tables S1 to S4 and Fig. S1 and S2. Download jcm.01703-21-s0001.pdf, PDF file, 0.4 MB [file jcm.01703-21-s0001.pdf]

Supp Table 1: Derived Variables

Pathogen	Conditions
**Bacteria**	
Aeromonas	Aeromonas
B.fragilis	B.fragilis
Campylobacter	Campylobacter.jejuni/coli Cq< 35<br>OR Campylobacter.pan Cq<35
Clostridium difficile	C.difficile Cq < 35
Enteroaggregative E coli (EAEC)	EAEC_aaiC Cq < 35<br>OR EAEC_aatA Cq < 35<br>OR EAEC_aggR Cq < 35
Enteropathogenic E coli (EPEC)	EPEC_bfpA Cq < 35<br>OR EPEC_eae Cq < 35
Typical EPEC	EPEC_bfpA Cq < 35<br>AND EPEC_eae Cq < 35
Atypical EPEC	EPEC_eae Cq < 35
Enterotoxigenic E coli (ETEC)	ETEC_STh Cq < 35<br>OR ETEC_STp Cq < 35<br>OR ETEC_LT Cq < 35
Helicobacter pylori (H. pylori)	H. pylori Cq <35
Mycobacterium tuberculosis (M.tb)	M.tb Cq<35
Plesiomonas	Plesiomonas Cq<35
Salmonella	Salmonella Cq<35
Shiga-toxin producing E. coli (STEC)	STEC_stx1 Cq < 35<br>OR STEC_stx2 Cq < 35
Shigella	Shigella/EIEC Cq < 35
Shigella sonnei	Shigella/EIEC Cq < 35<br>AND S. sonnei Cq < 35
Shigella flexneri 1b,2a, 2b,3a, other	Shigella/EIEC Cq < 35<br>AND S.flexneri_non6 Cq > 35 or ‘undetermined’<br>AND Shigella_Clade1 Cq < 35
Shigella flexneri 6	Shigella/EIEC Cq < 35<br>AND S. flexneri 6 Cq < 35
V.cholerae	V.cholerae Cq < 35
**Fungi**	
E.intestinalis	E.intestinalis Cq<35
E.bieneusi	E.bieneusi Cq<35
**Virus**	
Adenovirus	Adenovirus.40.41 Cq< 35<br>OR Adenovirus.pan Cq<35
Astrovirus	Astrovirus Cq<35
Norovirus	Norovirus GI Cq< 35<br>OR Norovirus GII Cq< 35
Rotavirus	Rotavirus_P4 Cq< 35<br>OR Rotavirus_P6 Cq< 35<br>OR Rotavirus_P8 Cq< 35<br>OR Rotavirus_G1 Cq< 35<br>OR Rotavirus_G2 Cq< 35<br>OR Rotavirus_G3 Cq< 35<br>OR Rotavirus_G4 Cq< 35<br>OR Rotavirus_G8 Cq< 35<br>OR Rotavirus_G9 Cq< 35<br>OR Rotavirus_G12 Cq< 35<br>OR Rotavirus Cq< 35

Sapovirus	Sapovirus Cq< 35
**Parasites**	
Ancyclostoma	Ancyclostoma Cq<35
Ascaris	Ascaris Cq<35
Blastocystis	Blastocystis Cq<35
Cyclospora	Cyclospora Cq<35
Cryptosporidium	Cryptosporidium.hominis Cq<35<br>OR Cryptosporidium.parvum Cq<35<br>OR Cryptosporidium Cq<35
Entamoeba	E.histolytica Cq<35<br>OR pan.Entamoeba Cq<35
Giardia	Giardia.A Cq<35<br>OR Giardia.B Cq<35<br>OR Giardia Cq<35
Hymenolepis nana (H.nana)	H.nana Cq<35
Isospora	Isospora Cq<35
Necator	Necator Cq<35
Schistosoma	Schistosoma Cq<35
Strongyloides	Strongyloides Cq<35
Trichuris	Trichuris Cq<35

Supp Table 1 presents the definition of derived variables used in the analysis.

Supp Table 2: Results from negative binomial regression to quantify risk factors for the number of pathogens present in each individual's stool

Risk factor	Ratio of expected number of pathogens	p value
Age group[1]		
3-5	0.87	0.09
6-17	0.91	0.23
Sex		
Female	1.07	0.33
Region[2]		
Littoral	0.90	0.23
Dysentery	1.37	<0.01
Non-acute Diarrhea	1.38	<0.01
Took any antibiotics before	1.19	0.27

[1] reference group: age 0-2 group

[2] reference group: Far North Region

Supp Table 3: Results from Logistic Regression to quantify risk factors for if there is highly diarrhea-associated pathogens:

Risk factor	Odds ratio	p value
Number of pathogens	1.44	< 0.001
Age group[1]		
3-5	1.50	0.18
6-17	1.56	0.14
Sex		
Male	1.63	<0.05
Region[2]		
Littoral	2.19	<0.01
Dysentery	0.87	0.74
Non-acute Diarrhea	1.26	0.57
Took any antibiotics before	1.08	0.89

[1] reference group: age 0-2 group

[2] reference group: Far North Region

Supp Table 4: CF type positivity by ETEC type positivity, per specimen

		ETEC type							
		STh only	STp only	LT only	LT+ STh+	LT+ STp+	STh+ STp+	LT+ STh+ STp+	Any ETEC
CF type	CFA.I	0	0	1	11	0	0	3	15
	CS1	0	0	1	5	1	0	5	12
	CS2	0	0	3	5	0	0	2	10
	CS3	0	0	1	8	0	0	2	11
	CS5	1	0	0	1	0	0	2	4
	CS6	1	4	5	3	1	2	7	23
	Any CF/CS*	1	4	8	22	2	2	8	47
	No CF/CS	3	0	45	7	5	0	0	60
	Total	4	4	53	29	7	2	8	107

Supp

*The total is not equal to the sum of CF types because some specimens had multiple CF types detected. (For example one stool sample that was positive for STh only also was positive for CS5 and CS6 resulting in the 1 sample marked as any CF/CS)

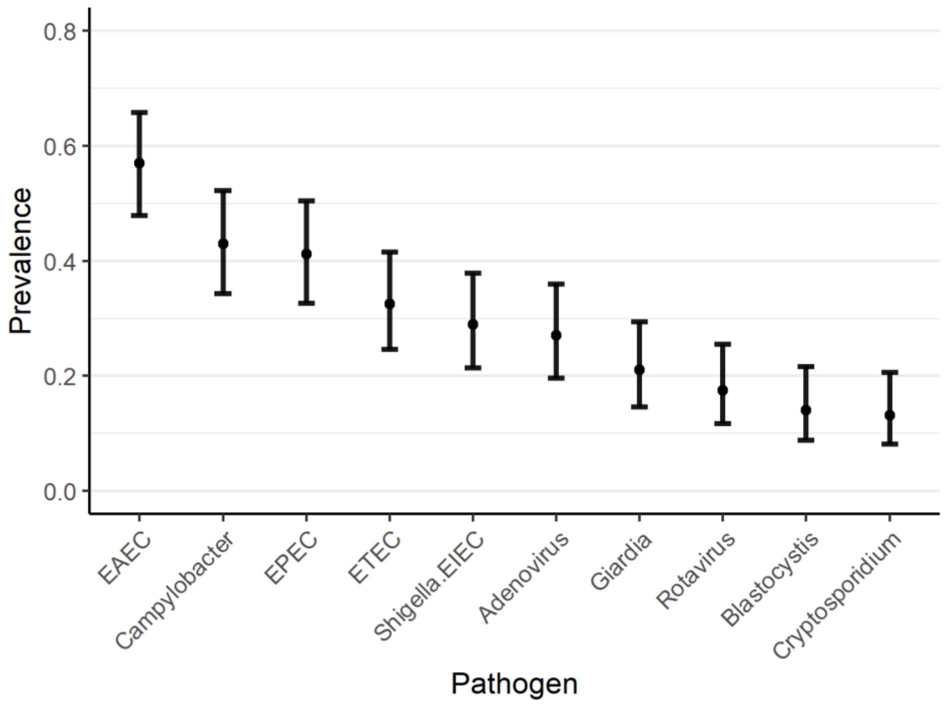

Supp Figure 1a: The prevalence of the top 10 pathogens, age group 0-2.

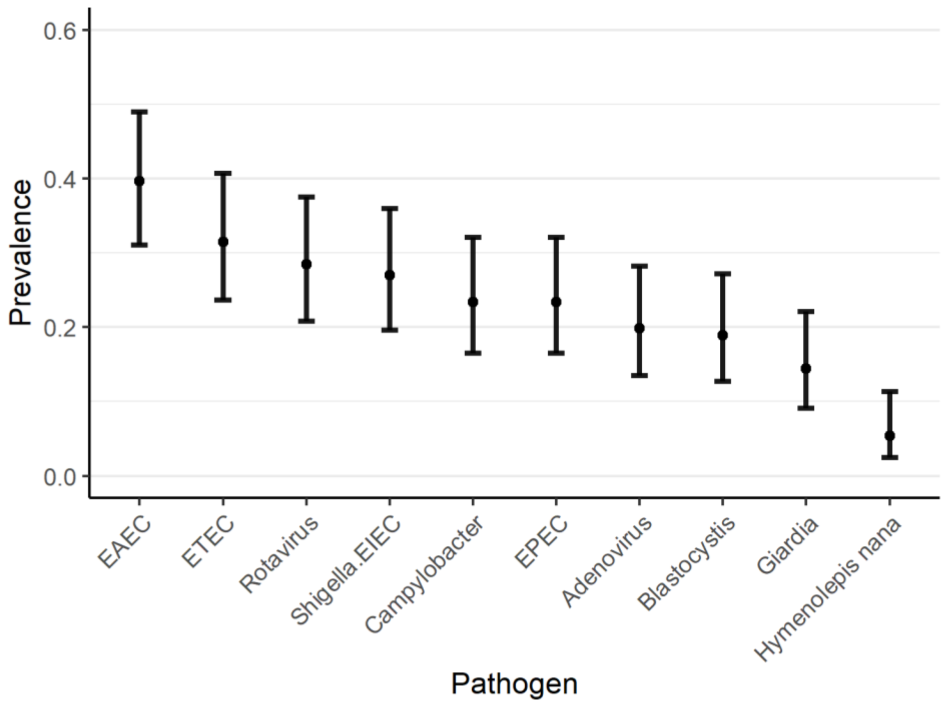

Supp Figure 1b: The prevalence of the top 10 pathogens, age group 3-5.

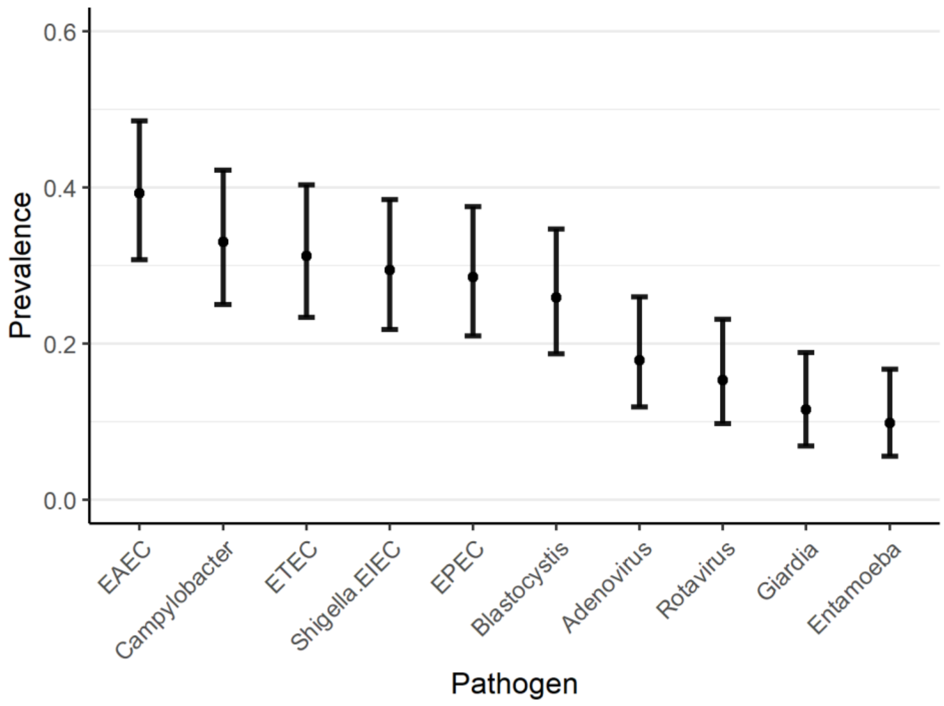

Supp Figure 1c: The prevalence of the top 10 pathogens, age group 6-17.

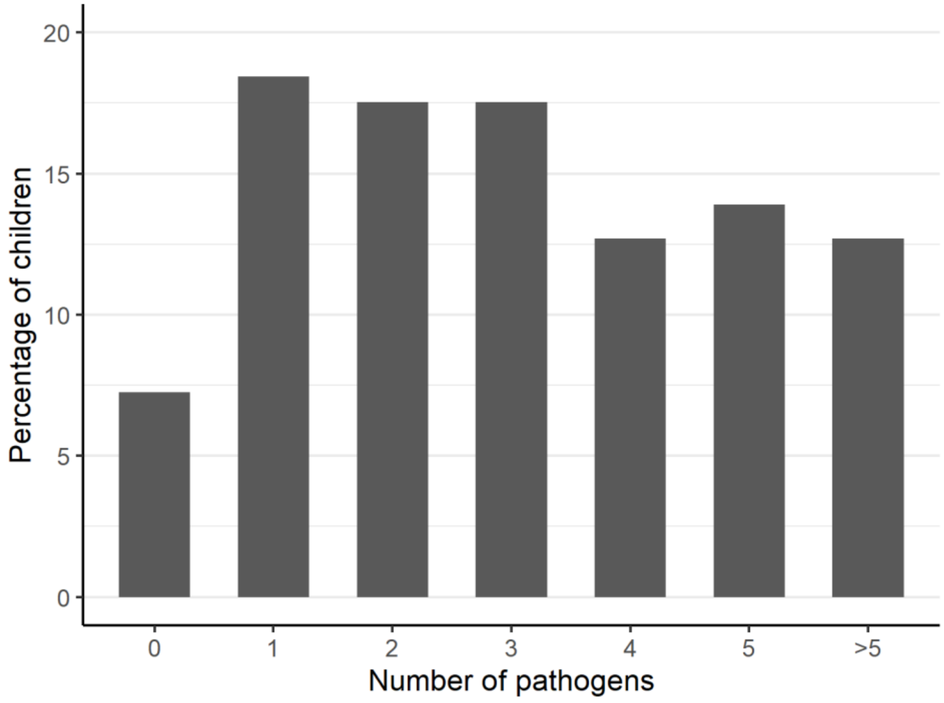

Supp Figure 2. Distribution of number of pathogens present in each individual’s stool
